# Diabetic Capital Punishment: Time for Amnesty

**DOI:** 10.3390/jcm11216562

**Published:** 2022-11-04

**Authors:** Raúl Juan Molines-Barroso, Mateo López-Moral, José Luis Lázaro-Martínez

**Affiliations:** Diabetic Foot Unit, Clínica Universitaria de Podología, Facultad de Enfermería, Fisioterapia y Podología, Universidad Complutense, Instituto de Investigación Sanitaria del Hospital Clínico San Carlos (IdISSC), 28040 Madrid, Spain

## Discussion

A study has shown that 19–34% of patients with diabetes will develop a foot ulcer in their lifetime [1], and close to 50% of these patients will die within 5 years [2,3]. A total of 22% of diabetes patients with ulcers are more likely to die than patients with diabetes who never develop ulceration [4].

Healthcare professionals have made significant efforts to treat ulcers. Recent findings show that ulcer healing time has been reduced, thereby lowering the associated complications [5,6]. However, it is estimated that 40% of patients will experience ulcer recurrence within 1 year after ulcer healing, almost 60% within 3 years, and 65% within 5 years. This issue consequently increases morbidity, reduces quality of life, and increases health care costs [7]. Thus, these patients are considered in a state of remission rather than being healed [1].

The “Pareto Principle”, also known as the 80/20 rule, states that 80% of outputs come from 20% of inputs [8]. In the context of health care, this rule suggests that 80% of resources are assigned to treat 20% of patients with ulceration, and 20% of the available resources are allocated to treat 80% of patients at high risk of ulceration. Thus, more implications by the industry must avoid the first and recurrent ulceration.

Each ulceration involves new debridement or surgical interventions, long-term immobilization, prolonged times hospitalizations, and multiple antibiotic treatments. Moreover, many patients may require partial amputation, contributing to increased morbidity and the risk of death [8]. As more amputation and surgical interventions are carried out for a patient, more biomechanical disturbances, and sometimes more difficulty in avoiding recurrent ulceration through conservative treatments, emerge, which can lead to a “hard-to-prevent” patient.

It is well-established that the primary and secondary preventions of foot ulcers are foot screening, stratifying patient categories based on the foot ulceration risk, the routine wearing of appropriate footwear, treating pre-ulcerative signs of foot, and providing patient and family education in foot self-care [9].

Several risk factors of ulceration have been identified, which include neuropathy, excessive plantar pressure, age, sex, BMI, or HbA_1c_ [10,11]. However, studies have shown a paucity of evidence regarding the predictive efficacy of the early diagnosis of symptoms [10,12]. Thus, developing models of clinical prediction, as well as the design and validation of high-quality randomized controlled trials (RCTs), is vital for identifying patients at high risk of ulceration across different health care settings [13].

Therapeutic footwear and the self-monitoring of foot skin temperatures are important interventions to prevent recurrent plantar diabetic foot ulcers [14]. These therapeutic strategies and the treatment of modifiable risk factors, such as regular callus removal, are vital for relapsing recurrent plantar ulcers [15]. However, a recent study has shown that patients have a low adherence to some of these preventive interventions, which may undermine the effectiveness of this approach [16]. The prevention of first ulcers and nonplantar ulcers has not been practically investigated [14].

Other interventions are sometimes widely applied. Although surgical interventions are recommended to reduce ulcer recurrence, studies have not demonstrated sufficient evidence to support the prevention of ulcer recurrence [14,15].

Surgical offloading is effective in managing plantar pressure and relieving the effect of the ulcer site. These treatments should be a proper indication in patients in remission because the efficacy of preventative treatments could be insufficient in 1 out of 4 patients at high risk of developing an ulcer [17,18]. Thus, surgical offloading is a treatment for recalcitrant ulcers [19]. After surgical offloading, recurrent ulceration at other sites is different from the initial ulceration, and this contributes to transferring syndrome [20]. Thus, comparative studies will benefit surgical decision-making remission in patients by avoiding recurrence, defining time points, and protecting against irretrievable tissue loss/re-ulceration through surgical offloading [19].

Patient and family education is vital for improving adherence to treatments, protecting the feet, increasing the implication of foot self-care, and identifying problems associated with prevention programs. However, patient education alone is not sufficient to prevent recurrence and amputation [21]. Thus, more integral structured programs of education are recommended as a tool for reducing the recurrence rates [14].

Adherence to prescribing treatments is vital for patients with a loss of protective sensation in the feet. Two RCTs with a very low risk of bias have shown that partial adherence results are insufficient in protecting high-risk patients from developing recurrent ulcers [17,18]. This problem is higher during patient daily life (when they are not observed in a RCT framework), and adherence to prevention measurements could be reduced. It has been reported that nearly 50% of patients do not use adequate footwear when they are outdoors [22], and nonadherence increases when patients are indoors. Suboptimal adherence is more evident over time (after 6 months of footwear use) [23]. Economic reasons or problems with design could explain suboptimal adherence in some patients [23]; however, the refusal to use footwear is also common, even among patients who receive footwear for free every 6 months. Unfortunately, the term “patient in remission” is not adopted by them, and this is considered a behavior that causes preventative measures to be neglected [24].

Since patient education has not been effective in reducing ulcer recurrence, it should be guided toward a model of identifying human response that cause harmful behavior in high-risk patients. Future studies should focus on how behavioral changes could be used to reduce recurrent ulceration. Moreover, educational programs should include these new findings to improve adherence. Furthermore, the industry should collaborate in the design and development of supplementary tools and computerized intelligent systems for the monitoring and surveillance of high-risk feet. More investigations are needed to identify models of high-risk patients and hard-to-prevent patients so that they may benefit from surgical off-loading. The goal is to reduce the recurrent ulceration without precipitate transfer lesions. These recommendations will resolve the lack of evidence related to the prevention of first foot ulcers and nonplantar foot ulcers. Further investigations must prevent “diabetic capital punishment”, which entails the first occurrence of ulcers in patients with diabetes.
